
JOURNAL INFORMATION
==============================
NLM Title Abbreviation: Clin Infect Dis
Journal ID: Clin Infect Dis
NLM Journal ID: 9203213
Journal ID: cid

Clinical Infectious Diseases: An Official Publication of the Infectious Diseases Society of America

ISSN: 1058-4838
EISSN: 1537-6591

ARTICLE INFORMATION
==============================
PMCID: PMC8500102
PMID: 34558608
DOI: 10.1093/cid/ciab855
Article ID: ciab855
Article version: 1
Subjects: Correspondence, AcademicSubjects/MED00290

WHO guidance to support HIV care models during the Covid-19 era

Ford Nathan
Vitoria Marco
Doherty Meg
Human Immunodeficiency Virus, Hepatitis, and Sexually Transmitted Infections Unit, World Health Organization , Geneva, Switzerland
Correspondence: N. Ford, Human Immunodeficiency Virus, Hepatitis, and Sexually Transmitted Infections Unit, World Health Organization, 1211 Geneva 27, Switzerland (fordn@who.int)
Electronic publication date: 2021 Sep 24
Electronic Location ID: ciab855
Received 2021 Sep 15
Copyright: © The Author(s) 2021. Published by Oxford University Press for the Infectious Diseases Society of America.
Copyright year: 2021
License: This is an Open Access article distributed under the terms of the Creative Commons Attribution License (https://creativecommons.org/licenses/by/4.0/), which permits unrestricted reuse, distribution, and reproduction in any medium, provided the original work is properly cited.
License URL: https://creativecommons.org/licenses/by/4.0/

article-lifecycle: PAP
edited-state: accepted-manuscript

==============================

### WHO guidance to support HIV care models during the Covid-19 era

**Nathan Ford, Marco Vitoria, Meg Doherty**

Human Immunodeficiency Virus, Hepatitis, and Sexually Transmitted Infections Unit, World Health Organization, Geneva, Switzerland

Correspondence: N. Ford, Human Immunodeficiency Virus, Hepatitis, and Sexually Transmitted Infections Unit, World Health Organization, 1211 Geneva 27, Switzerland (fordn@who.int)

Accepted Manuscript

© The Author(s) 2021. Published by Oxford University Press for the Infectious Diseases Society of America.
This is an Open Access article distributed under the terms of the Creative Commons Attribution License (https://creativecommons.org/licenses/by/4.0/), which permits unrestricted reuse, distribution, and reproduction in any medium, provided the original work is properly cited.

Dear editors –

We read with interest the recent Viewpoint summarizing guidelines launched by key HIV/AIDS societies to support HIV management during the COVID-19 pandemic.

In the first 6 months of the pandemic the World Health Organization (WHO) published three guidelines with the objective of ensuring continuity of essential health services, including HIV/AIDS care. In March 2020 WHO published operational guidance for maintaining essential health services during an outbreak. This was followed by guidance published in May 2020 which outlined recommendations to ensure continuity of select community-level health services, including expansion of HIV self-testing. In June 2020 WHO published operational guidance aimed at maintaining essential health services during the COVID-19 outbreak. This guidance recognized that health systems around the world are being challenged by increasing demand for care of people with COVID-19, compounded by fear, stigma, misinformation and limitations on movement that disrupt the delivery of health care for all conditions.

National surveys of 101 countries have revealed substantial disruption across all major health areas. HIV services have been disrupted in nearly half of countries, with 49% of countries reporting disruptions to HIV testing services and 43 (46%) countries reporting disruptions to HIV prevention services; 25% of countries reported disruptions to services to initiate new antiretroviral (ARV) treatment, and 17 (17%) reported disruptions to continuation of established ARV treatments. The Global Fund's results report 2021 confirms the devastating impact of COVID-19 on HIV services including a 16% decrease in TB treatment in PLHIV; 11% decline in prevention services and 22% reduction in HIV treatment initiation.

In September 2021 WHO released updated guidance on maintaining essential health services which provides direction on modifications and specific measures for safe delivery of HIV services, and considerations for transition towards restoration and recovery. Modelling suggests that in sub-Saharan African region a 6-month interruption of ART would result in an excess of over 500,000 adult HIV deaths and an up to two-fold increase in mother-to-child transmission of HIV. Additional modelling suggests that maintaining HIV services could lead to additional COVID-19 deaths; however the HIV-related deaths averted by providing services is at 100 times greater than the COVID-19 deaths averted by closing services.

Poorer quality clinical care due to over-burdened health workers and facilities, interruptions of the supply of other drugs and suspension of HIV testing would also have significant population impacts. To limit the potential for disrupted supply of ART and other essential medications and limit client volumes in health facilities, WHO recommends drug dispensing for ART should be for longer periods of up to 6 months. Table 1 summarizes WHO guidance on measures for safe delivery of HIV treatment and monitoring.

Accepted Manuscript

Restriction in movements has limited community-based care provision, while care in health facilities has been limited as staff have been diverted to the COVID-19 response. Many countries have relaxed movement restrictions or are cycling between restrictions and relaxations. Where restrictions on movement and the epidemiological situation permits, catch-up campaigns should be implemented to improve coverage of testing, prevention, and treatment interventions. WHO recommends that national programmes develop guidance for providers on how best to successfully locate and re-enrol clients into care, especially if their treatment has been interrupted. This should include reducing existing operational, regulatory, legal, and structural barriers that impede the expansion of essential services for HIV and related infectious diseases such as viral hepatitis and sexually transmitted infections.

According to patient-level clinical data from 37 countries HIV infection is independently associated with a 30% increased risk of death among patients hospitalized with suspected or confirmed SARS-CoV-2 infection.  WHO has encouraged all PLHIV to be vaccinated and recommend countries to consider including them as a priority group for early COVID-19 vaccination in their national plans, particularly those with advanced HIV disease and co-morbidities. WHO is also monitoring the safety and efficacy of COVID-19 vaccines in PLHIV in ongoing trials.

Accepted Manuscript

**Funding**

This work was supported by a grant from the Bill and Melinda Gates Foundation.

Accepted Manuscript

### References

1. Guaraldi G, Milic J, Martinez E, et al. Human Immunodeficiency Virus (HIV) Care Models During the Coronavirus Disease 2019 (COVID-19) Era. *Clin Infect Dis* 2021; **73**(5): e1222-e7.
2. Operational guidance for maintaining essential health services during an outbreak. World Health Organization, March 25, 2020. https://apps.who.int/iris/handle/10665/331561.
3. Community-based health care, including outreach and campaigns, in the context of the COVID-19 pandemic. IFRC, WHO, UNICEF, May 2020. https://www.who.int/publications/i/item/WHO-2019-nCoV-Comm_health_care-2020.1.
4. Maintaining essential health services: operational guidance for the COVID-19 context interim guidance. World Health Organization, Geneva, 2020. https://www.who.int/publications/i/item/WHO-2019-nCoV-essential_health_services-2020.2
5. Second round of the national pulse survey on continuity of essential health services during the COVID-19 pandemic: January-March 2021. World Health Organization, Geneve, April 2021. https://www.who.int/publications/i/item/WHO-2019-nCoV-EHS-continuity-survey-2021.1.
6. Results Report 2021. The Global Fund, Geneva. https://www.theglobalfund.org/media/11304/corporate_2021resultsreport_report_en.pdf.
7. Jewell BL, Mudimu E, Stover J, et al. Potential effects of disruption to HIV programmes in sub-Saharan Africa caused by COVID-19: results from multiple mathematical models. *Lancet HIV* 2020; **7**(9): e629-e40.
8. Stover J KS, Mudimu E, Green D, Smith T, Taramusi I, et al. . The Risks and Benefits of Providing HIV Services during the COVID-19 Pandemic. MedRxiv, 03 March 2021.
9. Coronavirus disease (COVID-19): HIV and antiretrovirals. World Health Organization, Geneva, 30 November 2020. https://www.who.int/news-room/q-a-detail/coronavirus-disease-covid-19-hiv-and-antiretrovirals.
10. Clinical features and prognostic factors of COVID-19 in people living with HIV hospitalized with suspected or confirmed SARS-CoV-2 infection. WHO Global Clinical Platform for COVID-19. Geneva, 15 July 2021. https://apps.who.int/iris/bitstream/handle/10665/342697/WHO-2019-nCoV-Clinical-HIV-2021.1-eng.pdf.
11. Coronavirus disease (COVID-19): COVID-19 vaccines and people living with HIV. World Health Organization, Geneva, 14 July 2021. https://www.who.int/news-room/q-a-detail/coronavirus-disease-(covid-19)-covid-19-vaccines-and-people-living-with-hiv.

Accepted Manuscript

**Table 1: HIV treatment and monitoring**

| **Usual programme activities** | **Needed context modifications and specific measures for safe delivery of services** | **Considerations for transition towards restoration and recovery** |
|---|---|---|
| Routine screening for people with HIV | Use point-of-care CD4 cell count at ART start and return to care to diagnose<br><br>advanced HIV disease. | |
| Prevention of<br><br>Common<br><br>comorbidities | Modify prescribing to increase patients' supplies of TB preventive treatment<br><br>(isoniazid, 3HP, 1HP), sulfamethoxazole + trimethoprim, and fluconazole. | Multi month provision.<br><br>Telemedicine.<br><br>Safe drop-in service for symptomatic patients. |
| Routine ART: adults and adolescents | Emphasize same day ART start, including out of facility ART start (eg outreach and mobile services) according to context.<br><br>Extend to 6 monthly dispensing and promote community dispensing points. | Telemonitoring of recently started treatments. Online appointment scheduling.<br><br>Maintain emphasis in same day ART start, including out of facility ART start<br><br>Continue and expand group adherence support (small group, virtual, mobile phone format).<br><br>Improve community-based |

Accepted Manuscript

| | | |
|---|---|---|
| | | strategies to improve retention, adherence, and viral suppression in coordination with CBOs. |
| Routine ART: children | Prioritize optimal regimens containing RAL or DTG, and switch to 3 months dispensing for children >3 years of age.<br><br>For children <3 years: estimate if dose adjustment required before next visit and prescribe accordingly<br><br>Follow-up via phone, SMS or virtual /tele consult together with caregiver (including for planned dose adjustment)<br><br>If insufficient stocks deliver ARVs at home/community. | Do catch up campaigns for early infant diagnosis (EID) and ART initiation at first vaccination or other well child visits if missed. |
| HIV: Adherence and retention in care | Emphasize e/M-health strategies.<br><br>Establish tele-modalities for individual counselling that adhere to the principles of confidentiality and non-coercive decisions with adolescents. | Maintain tele-modalities.<br><br>Scale up differentiated service delivery models, such as peer/group counselling for adherence support and tracing and re-engagement of people who have disengaged, and community-based strategies to improve retention, adherence, and viral suppression in coordination with CBOs. Online appointment scheduling. |
| TB-HIV co-infection | Provide adequate stocks of TB medicines to all patients to take home to ensure treatment completion without having to visit treatment centres unnecessarily to collect medicines.<br><br>Use e/M-health adherence support. | Implement catch-up campaigns<br><br>for initiation of TB preventive<br><br>treatment if delayed or missed. |
| Preventives and diagnostics for patients with advanced HIV disease | Modify to promote out-of-clinic delivery of elements of advanced disease<br><br>package of care (prophylaxis, and screening for CD4 count and cryptococcal | Full clinical check-up. Safe drop-in circuits for symptomatic patients. |

Accepted Manuscript

| | | |
|---|---|---|
| | antigen, and TB screening for lipoarabinomannan antigen). Consider satellite care centres for PLHIV with advanced disease.<br><br>Reinforce vaccination against influenza.<br><br>Maintain regular follow up through distanced clinic checkup or e/m health. | |
| ART monitoring | Reduce viral load testing to every 12 months unless clinically indicated. | Catch up viral load campaign. |

Accepted Manuscript
